# Intraosseous Cavernous Hemangioma: A Rare Presentation in Maxilla

**Published:** 2014-09-23

**Authors:** Burak Kaya, Servet Elçin Işılgan, Cem Çerkez, Volkan Otrakçı, Savaş Serel

**Affiliations:** ^a^Department of Plastic Reconstructive and Aesthetic Surgery, Ankara University School of Medicine, Ankara, Turkey; ^b^Girne Doktor Akçiçek Hospital, Clinic of Plastic and Reconstructive Surgery, Girne, TRNC, Cyprus; ^c^Diyarbakır Training and Research Hospital, Clinic of Plastic Reconstructive and Aesthetic Surgery, Diyarbakır, Turkey

**Keywords:** cavernous hemangioma, intraosseous, Medpor, maxilla, orbital protrusion

## Abstract

**Objective:** Hemangiomas are benign vascular lesions mostly arising from soft tissues. However, intraosseous hemangioma is a rare entity, comprising only 1% of benign bone tumors. We describe here the presentation, diagnosis, and treatment of a 42-year-old woman with a painless hard swelling—diagnosed to be intraosseous hemangioma via orbital magnetic resonance imaging—localized on the left infraorbital margin. **Methods:** After en bloc excision of the mass with safety margins through a subciliary incision, the defect was reconstructed via Medpor, which was fixed to the drilled bones with polypropylene sutures. **Results:** Histopathological diagnosis of the specimen revealed intraosseous cavernous hemangioma. There seems to be no recurrence or any cosmetic deformity 3 months postoperatively. **Conclusion:** Total surgical excision is the preferred method of treatment for intraosseous hemangiomas with reconstruction. In this patient, we used Medpor for reconstruction of orbital floor, the infraorbital orbital rim and anterior wall of maxillary sinus. We think that Medpor is a good option since a natural smooth malar contour and adequate orbital rim can be achieved and there is no any other scar.

Intraosseous vascular anomalies are rare and account for less than 1% of all bony tumors. The sites most commonly involved are the vertebral column and the skull. Within the calvarium, the parietal bone is most commonly involved followed by the frontal bone. Within the facial skeleton, vascular anomalies occur in the mandible, maxilla, and nasal bones.^1^ We report a case of intraosseous cavernous hemangioma of the maxilla of the left orbital floor, protruding toward the left maxillary sinus and orbital cavity.

## METHODS/CASE PRESENTATION

A 42-year-old woman was referred to our clinic with a complaint of gradually enlarging mass on her left cheek that she had noted in the last 3 years. The mass was painless and did not produce any symptom except for a hint of cosmetic deformity. On physical examination, a bony, hard, painless mass measuring about 1.5 × 1 cm^2^ was palpated on the left infraorbital margin. There were no signs in the eye and in particular any abnormality in vision, ocular movement, or position of the globe; there was no regional paresthesia. The overlying skin was mobile and normal in appearance. No other specific finding was observed in her head or neck region.

**Figure 1:**
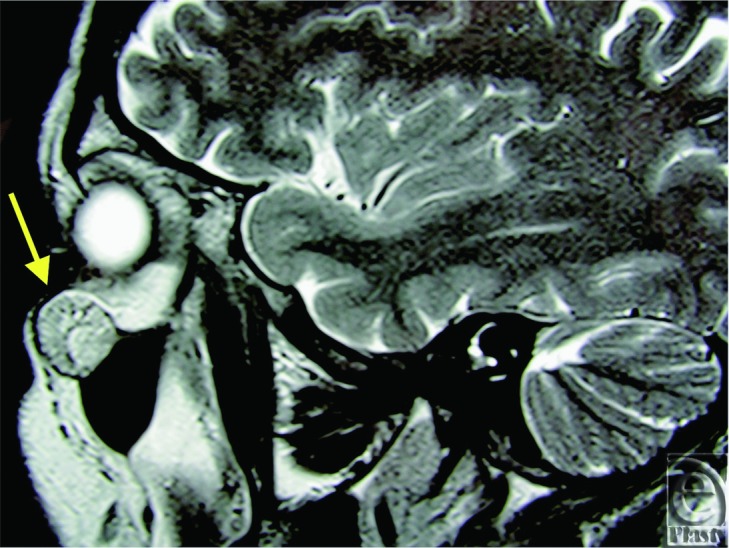
Sagittal MRI image of the infraorbital lesion.

**Figure 2:**
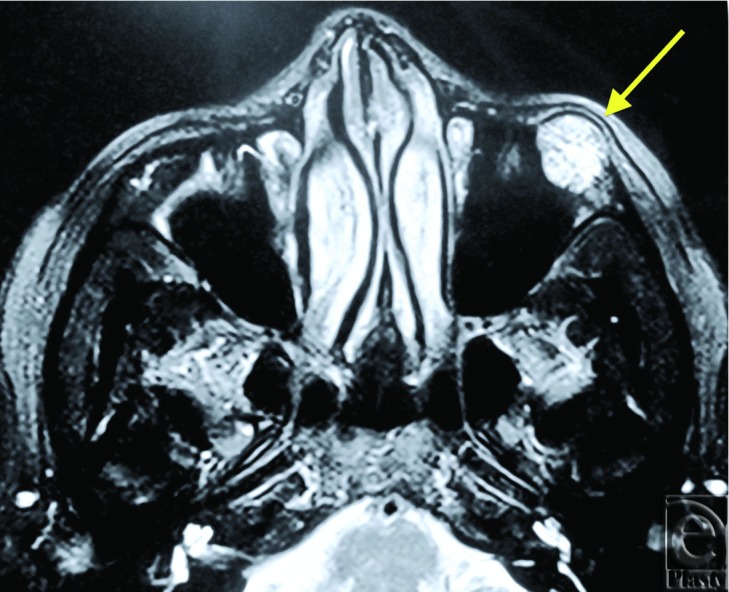
Axial MRI image of the infraorbital lesion.

An orbital magnetic resonance imaging (MRI) showed a 16-mm diameter, well-circumscribed mass originating from left maxilla in close proximity to the zygomatic bone (Fig 1). The mass was on the left inferolateral orbital wall protruding toward orbital cavity and maxillary sinus (Fig 2). Intraorbital structures were reported to be intact without any sign of infiltration, compression, or displacement. The diagnosis was intraosseous cavernous hemangioma. Eosinophilic granuloma and fibrous dysplasia was considered in the differential diagnosis.

En bloc excision was decided to be the right surgical approach. Preoperative routine blood investigations were within normal limits. Anesthesiology consultation was done and the patient was scheduled for surgery under general anesthesia.

## RESULTS

**Figure 3:**
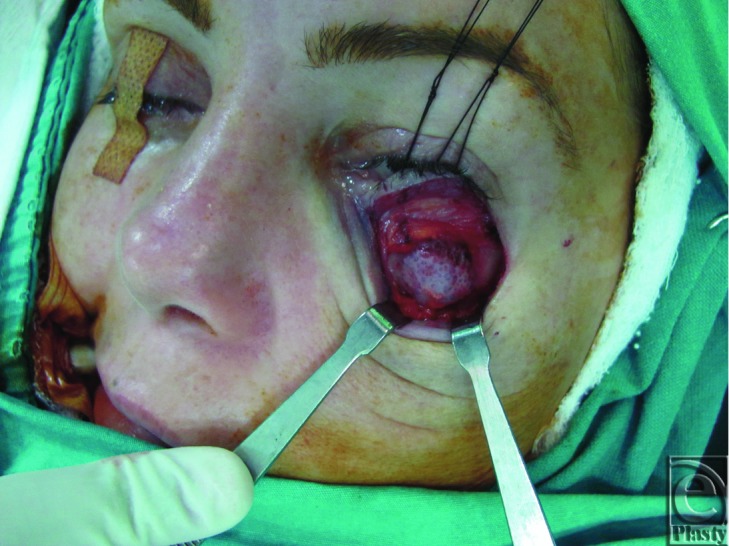
Intraoperative appearance of the mass being exposed.

**Figure 4:**
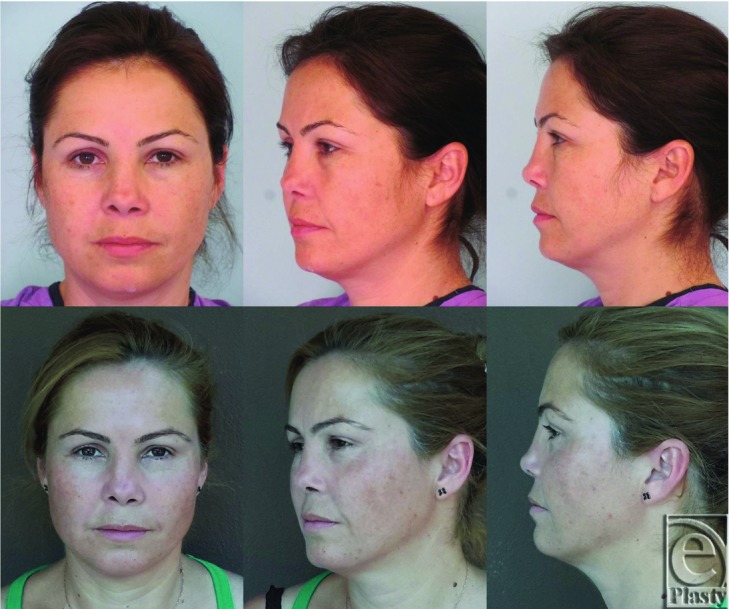
Upper row: Preoperative photographs of the patient. Lower row: Postoperative photographs of the patient.

A subciliary incision was performed after local anesthesia infiltration. Periosteal flap was raised, and mass exposed (Fig 3). The mass extended from the left infraorbital foramen to orbital floor. After complete excision of the mass with safety margins, a hollow was encountered on the anterior maxillary wall and the orbital floor that measured about 1.5 × 1 × 2.5 cm^3^. After irrigation of the left maxillary sinus and hemostasis by bone wax, the defect was reconstructed via Medpor, which was fixed to the drilled bones with polypropylene sutures. On gross examination, the mass was a characteristically purplish-red with honeycomb appearance (Fig 3). Histopathological diagnosis of the specimen revealed intraosseous cavernous hemangioma. There seems to be no recurrence or any cosmetic deformity 28 months postoperatively (Fig 4).

## DISCUSSION

Hemangiomas are benign vascular lesions that are typically identified during infancy and are known to regress by adolescence.^2^ Although head and neck region is not an uncommon localization for hemangiomas, most arise from soft tissues. Intraosseous hemangiomas are very rare, slow-growing benign vascular anomalies accounting for 1% of all benign bone tumors. The most frequent sites of involvement are calvarium and vertebral column.^3^ The maxillofacial involvement is rare and mandible, maxilla, and nasal bones are the most frequently affected sites, respectively.^2^^,^^3^

They are classified as cavernous or capillary type according to their vascular network histopathologically. The cavernous hemangioma is composed of large thin-walled vessels and sinusoids lined with a single layer of endothelium. However, a small fine vascular network filled with blood forms the capillary hemangioma. Capillary hemangiomas are usually present at birth. In contrast, most cavernous hemangiomas occur in adulthood. Almost all intraosseous hemangiomas of the facial skeleton are to be cavernous type.^4^ The differential diagnoses for intraosseous cavernous hemangioma include fibrous dysplasia, osteoma, Langerhans cell histiocytosis, dermoid tumor, and multiple myeloma.

Women in the fourth and fifth decades of life are mostly affected.^5^ Although the cause of intraosseous hemangioma is still uncertain, local trauma is thought to be one possible factor. In our patient, there was no such predisposition for intraosseous hemangioma development. As intraosseous hemangioma tends to grow very slowly, it remains clinically silent until the tumor becomes large. Therefore, early detection is crucial to a lesser cosmetic deformity.

Computed tomography is considered the most useful imaging technique because of its unique characterization of trabecular and cortical details, showing the honeycomb appearance.^1^ MRI provides information about any associated soft tissue element. In our case, close proximity of the mass to the orbit made MRI compulsory.

The goal of the treatment in hemangioma is to remove the tumor completely without any functional deficit, cosmetic deformity, or significant tissue loss. Biopsy of the lesion in order to exclude malignancy should be done cautiously because of the risk of severe bleeding.^6^

In the past, radiotherapy and sclerotherapy were the treatment of choice. But today radiotherapy may only be reserved for cases in which surgery is not feasible due to the adverse effects such as tissue necrosis, retardation of growth of bones and teeth, telangiectasia, and malignant degeneration, and sclerosing agents are used for soft-tissue hemangiomas of the head and neck. Other treatment modalities include angiography with embolization, curettage, and cryotherapy.^7^^-^10

Total surgical excision is the preferred method of treatment for intraosseous hemangiomas with reconstruction. Depending on the size of the defect and the surgeon preferences, autologous or heterologous materials can be used. Some techniques include hydroxyapatite associated with titanium mesh for reconstruction,^11^ Medpor,^12^ and bone grafting.^13^ In our patient, Medpor was preferred to reconstruct orbital floor, the infraorbital orbital rim, and anterior wall of maxillary sinus because the patient would not accept any other scar. The result has been satisfactory, achieving a natural smooth malar contour and adequate orbital rim.
